# Echocardiographic diagnosis of anomalous pulmonary venous connections: Experience of 84 cases from 1 medical center

**DOI:** 10.1097/MD.0000000000005389

**Published:** 2016-11-04

**Authors:** Ziming Zhang, Li Zhang, Feng Xie, Bing Wang, Zhengxing Sun, Shuangshuang Kong, Xinfang Wang, Nianguo Dong, Guohua Wang, Qing Lv, Yuman Li, Ling Li, Mingxing Xie

**Affiliations:** a Department of Ultrasound, Union Hospital, Tongji Medical College, Huazhong University of Science and Technology, Wuhan, China; b Department of Cardiovascular Medicine, University of Nebraska Medical Center, Omaha, NE; c Department of Cardiovascular Surgery, Union Hospital, Tongji Medical College, Huazhong University of Science and Technology, Wuhan, China.

**Keywords:** APVC, cardiac surgery, congenital malformation, PAPVC, TAPVC, ultrasonography

## Abstract

We sought to evaluate the value of echocardiography in the diagnosis of different types of anomalous pulmonary venous connections (APVCs) and summarize the diagnostic experience. A total of 84 patients with APVC were confirmed by surgery (n = 82) or computerized tomography angiography (CTA) (n = 2) in the last 6 years (2008–2014) at the Wuhan Union Hospital. The total anomalous pulmonary venous connection (TAPVC) cases account for 60.7%, and partial anomalous pulmonary venous connection (PAPVC) cases account for 39.3% among the 84 cases that were identified. The 51 TAPVCs were classified by the Darling method—type I (41.1%), type II (52.9%), type III (1.9%), and type IV (3.9%). The most common drainage path of type I was common pulmonary drainage to the left innominate vein via vertical vein, and the coronary sinus drainage was the most common path in type II. Compared with surgical or CTA results, the sensitivity and specificity of echocardiography in the diagnosis of APVCs were 97.6% and 99.9%, respectively. The echocardiography misdiagnoses were mainly seen in PAPVCs. Of the TAPVCs and PAPVCs correctly diagnosed by echocardiography, the diagnostic accuracy of classification were 94% and 100%, respectively. Echocardiography has specific value in diagnosing and classification of APVC, especially the supracardiac and cardiac TAPVCs. Multiplane scan views and color Doppler improve the display of drainage pathway.

## Abstract 2

Supplemental Digital Content is available in the text

## 1 Introduction

Anomalous pulmonary venous connection (APVC) is an uncommon congenital anomaly in which pulmonary venous blood flows directly into the right side of the heart or into the systemic veins. The venous abnormality may be partial or total. Total anomalous pulmonary venous connections (TAPVCs) represent approximately 1% to 3% of major congenital heart diseases.^[1,2]^ The overall incidence of partial anomalous pulmonary venous connections (PAPVCs) is approximately 0.5%.^[3]^ Echocardiography is regarded as the preferred initial screening and diagnostic modality in patients with APVC.^[4]^ The purpose of our study was to compare echocardiographic features and surgical or computerized tomography angiography (CTA) results of consecutive APVC patients over the last 6 years at the Union Hospital, Huazhong University of Science and Technology, to present the imaging findings of different types of APVCs and to evaluate the diagnostic value of echocardiography in APVCs.

## 2 Methods

### 2.1 Clinical data (study population)

The study was approved by the local research ethics committee at Tongji Medical College, Huazhong University of Science and Technology, China. All procedures were performed as part of routine care and testing and not specifically for the purpose of this study. All of data used were anonymized as all patients enrolled were identified by a progressive number. Each study participant provided written informed consent to publish these case details. All of the procedures and data analysis were performed by the authors in accordance with the approved guidelines; specific contributions of all the enlisted authors are provided below. A total of 84 patients with APVC were confirmed by surgery or CTA from March, 2008 to February, 2014 at the Union Hospital. The surgical cases account for 0.7% of all open-heart operations during this period. The patient group consisted of 46 males and 38 females whose ages ranged from 23 days to 55 years. Among those with the TAPVC type, 36 cases were less than 1 year old, and 5 cases were older than 10 years. Among those with the PAPVC type, 5 cases were less than 1 year old, and 14 cases were older than 10 years. The clinical characteristics of all APVC patients are shown in Table 1.

**Table 1:**
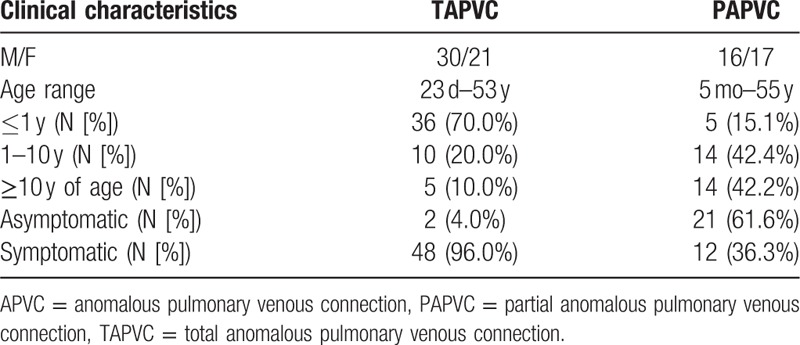
Clinical data of 84 APVC patients.

### 2.2 Echocardiography

All patients were evaluated by transthoracic 2-dimensional echocardiography and Doppler echocardiography using a cardiac ultrasound system (Philips iE 33, Andover, MA; GE Vivid 7, Fairfield, CT; or Vingmed, Horten, Norway) with a 2.0- to 5.0-MHz probe. The patients were in the left lateral position, and multiple views were used to observe the pulmonary veins: parasternal, suprasternal, subcostal, apical, and some nonstandard views. The following details were identified: the existence of an APVC, the drainage site and course of the anomalous pulmonary veins (APVs), the existence of obstruction in the course, and the associated cardiovascular lesions. These echocardiographic characteristics were compared with the operative findings or CTA results.

### 2.3 Classification

#### 2.3.1 TAPVC

TAPVCs are described as supracardiac, cardiac, infracardiac, or mixed depending on the site or sites of connection.

In type I TAPVC (supracardiac), anomalous connection was at the supracardiac level, and connection to the left innominate vein (LIV) is found frequently. Other less-common supracardiac venous connections include the superior vena cava (SVC) and azygos veins (AZs).

In type II TAPVC (cardiac), the APVs connect to the heart, either to the right atrium (RA) directly or to the coronary sinus (CS).

In type III TAPVC (infracardiac), the anomalous connection occurs at the infracardiac level below the diaphragm and drains into the portal veins, ductus venous, hepatic veins, or inferior vena cava (IVC).

In type IV TAPVC (mixed pattern), the pulmonary veins drain to at least 2 different locations.

#### 2.3.2 PAPVC

According to the origins of APV, there are 3 different types: APVs originating from the left lung, from the right lung, or from both the lungs.

According to the drainage pathway of APV, the anomalous connection occurs at supracardiac, cardiac, or infracardiac level.

## 3 Results

The comparison of the echocardiographic and surgical or CTA results of the 84 APVC patients is listed in Tables 2 and 3. Among the 84 identified cases, we missed 2 cases by echocardiography which were PAPVCs detected after atrial septal defect (ASD) repair operation. Among the 82 cases diagnosed as APVC by echocardiography, 1 case of TAPVC was misdiagnosed as a PAPVC.

**Table 2:**
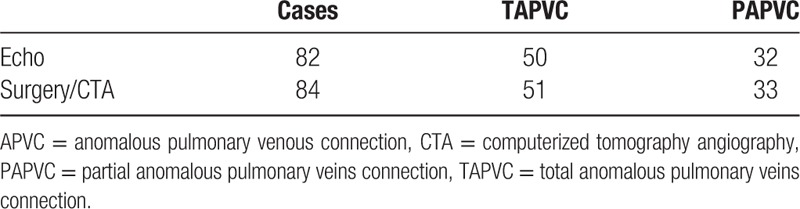
Comparison of the results of echocardiography and surgery/CTA for the diagnosis of an APVC.

**Table 3:**
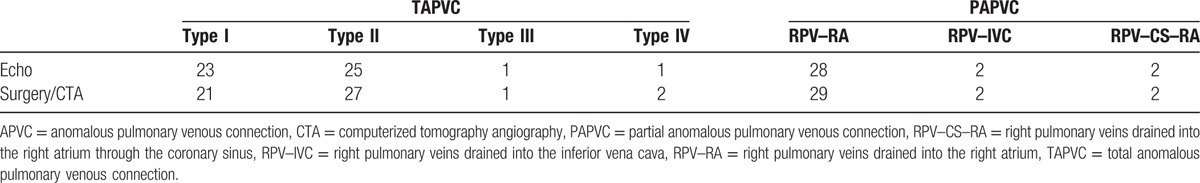
Comparison of the results of echocardiography and surgery/CTA for diagnosis of different drainage pathways of APVCs.

### 3.1 Distribution of the 84 APVC cases

#### 3.1.1 Classification of PAVC

The TAPVC cases account for 60.7% (51 cases) and PAPVC cases account for 39.3% (33 cases) of all 84 cases. The 51 TAPVCs were classified into 21 cases of type I (41.1%), 27 cases of type II (52.9%), 2 cases of type IV (3.9%), and 1 case of type III (1.9%). Of the 21 cases of type I, the common pulmonary vein (CPV) drainage to the LIV via the vertical vein (VV) occurred in 18 patients (85.7%), direct connection to the right SVC (RSVC) occurred in 2 patients (9.5%), and connection to the left SVC (LSVC) occurred in 1 patient. Of the 27 cases of type II, pulmonary vein drainage at the level of the CS occurred in 24 cases (88.8%), and connection to the RA occurred in 3 cases (11.1%).

There were 3 different drainage pathways in the 33 PAPVC cases: drainage of the right pulmonary veins (RPVs) into the RA occurred in 29 cases, drainage into the IVC showed in 2 cases, and drainage into the CS occurred in 2 cases.

#### 3.1.2 Obstruction of the drainage course

Obstruction of the VVs occurred in 3 cases of type I TAPVC. In 2 cases, the obstruction was located in the VVs, and in 1 case obstruction occurred in the opening to the RSVC.

#### 3.1.3 Associated cardiovascular lesions

Twelve types of associated lesions were found in these APVC cases. The most common lesions were ASD or patent foramen ovale (PFO), patent ductus arteriosus, and other lesions including atrioventricular septal defect (AVSD), the heterotaxy syndrome (HS), cor triatriatum single atrium (SA), and single ventricle. The most common associated cardiac malformation of PAPVC observed was the sinus venosus atrial septal defect (SVASD). The associated congenital cardiac malformations of APVC are shown in Table 4.

**Table 4:**
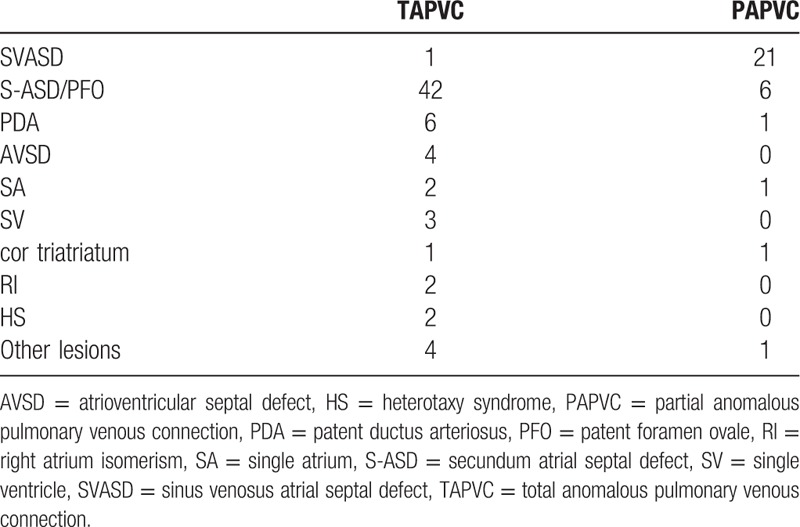
Associated congenital cardiac malformations of TAPVC and PAPVC.

#### 3.1.4 Diagnostic accuracy of echocardiography

The comparisons of the echocardiographic and surgical or CTA results in diagnosing APVCs are shown in Table 5. Compared with the surgical or CTA findings, the sensitivity and specificity of echocardiography in the diagnosis of APVCs were 97.6% and 99.9%. The echocardiography misdiagnoses were mainly observed in PAPVCs. Of the PAPVCs identified by surgery, 2 cases were missed by echocardiography and detected after ASD repair operations. In addition to the missed cases, the diagnostic accuracy of typing either TAPVC or PAPVC was 98.7% (81/82). Of the 50 cases of TAPVCs, the diagnostic accuracy of classification was 94% (47/50). Of the 32 PAPVCs diagnosed by echocardiography, the diagnostic accuracy of classification was 100%.

**Table 5:**
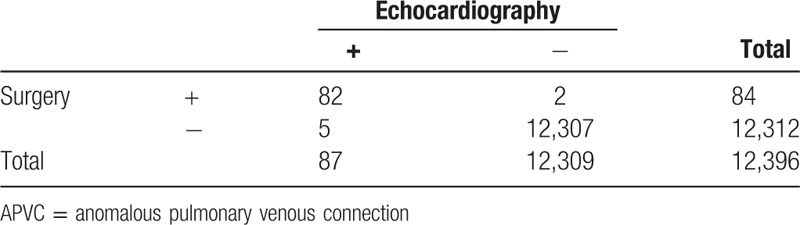
Diagnostic accuracy of echocardiography in APVC.

### 3.2 Echocardiographic features of TAPVC

#### 3.2.1 Supracardiac TAPVC

**Figure 1:**
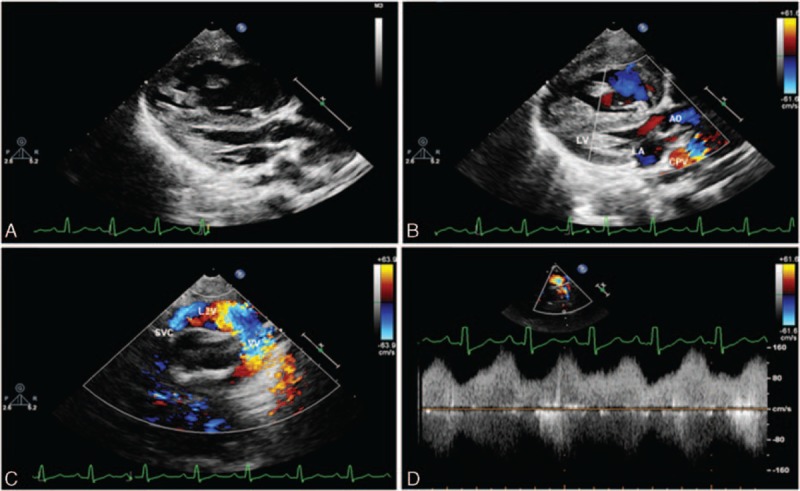
Transthoracic echocardiography shows supracardiac total anomalous pulmonary venous connection drainage to superior vena cava (SVC) via left innominate vein (LIV) of a 6-month-old boy. (A) Two-dimensional view of the parasternal long axis shows a small left side of heart, hypertrophy of right ventricle, and the common pulmonary vein (CPV) behind the left atrium (LA). (B) Color Doppler shows the flow in the CPV and no flow spray from CPV to LA. (C) Suprasternal view—the flow from CPV to SVC via vertical vein (VV) and LIV shows clearly by color Doppler. (D) Pulse Doppler shows the phasic flow pattern in VV, varying with the cardiac cycle.

There were 21 cases identified as having a supracardiac TAPVC. Three different drainage pathways were found in our cases. The most common pathway was the CPV drains into the LIV through the VV (CPV–VV–LIV). The suprasternal long axis and its rotational views are most useful for detecting this type of supracardiac TAPVC. A tubular structure that is a VV can be visualized beside the short-axis view of the aorta, and, by moving the transducer, it can be traced into the LIV, accompanied by enlargement of the LIV and SVC. Pulsed and color Doppler were further used to display the flow direction, velocity, and phasicity of the cardiac and respiratory cycles (Supplementary video 1). Color Doppler showed that the direction of the flow in the VV is counter to the flow in the aorta and in the left SVC, if it existed (Fig. 1).

**Figure 2:**
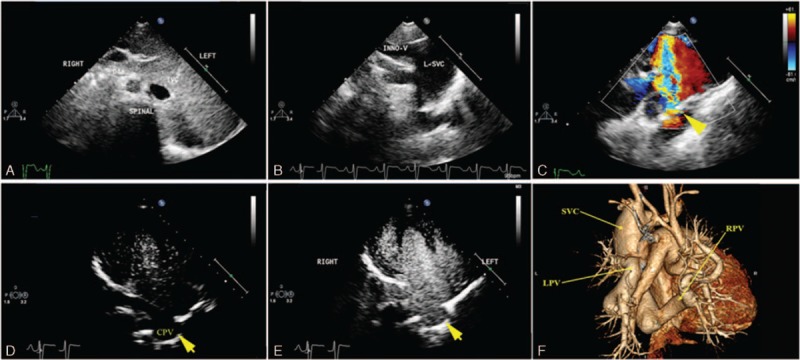
A 9-year-old girl with dextrocardia with anomalous pulmonary venous connections draining to aneurysmal left superior vena cava (LSVC) diagnosed by echocardiography, contrast echocardiography, and computerized tomography angiography (CTA). (A) The subcostal view shows the inferior vena cava located on the left side of the descending aorta. (B) The suprasternal view shows the LSVC dilated and connected to the innominate vein. (C) Using color flow Doppler, an abnormal accelerated flow spray into the LSVC was visualized. (D) Left-sided contrast shows no bubbles in the tubular structure (common pulmonary vein [CPV]) behind the atrium at first cardiac cycle. (E) After several cycles, bubbles appear in the CPV. (F) CTA shows the right atrium isomerism and left pulmonary vein confluence draining to the aneurysmal superior vena cava.

In patients with APVs directly connected to the SVC, the VV could not be found, and the openings to the SVC could not be easily detected by 2-dimensional echocardiography. The enlargement of the SVC and the sudden accelerated flow into the SVC, which is detected by color Doppler, can help to diagnose TAPVCs of this type. In the study, the pulmonary vein drained directly into the right SVC in 2 cases, and the pulmonary vein drained into the LSVC in 1 case. The diameter of SVC of these cases ranged from 2.8 to 3.3 cm wider than the normal-caliber SVC.^[5]^ Furthermore, an aneurismal dilatation of the LSVC measuring 5.6 cm in diameter was detected in the patient with pulmonary vein draining to LSVC, findings that were confirmed by CTA (Fig. 2). Echocardiography showed a symmetric midline liver, dextrocardia, right atrial isomerism, complete AVSD, double SVC, and TAPVC in this patient. Pulmonary vein openings could not be detected in the left atrium (LA) via the parasternal and apical 4-chamber views. Utilizing the suprasternal view, the RSVC and aneurysmal-dilated LSVC were detected. Using color flow Doppler, an abnormal accelerated flow spray into the LSVC was visualized (Supplementary video 2). The flow direction was opposite to that in the descending aorta. Tracing the LSVC by 2-dimensional echocardiography, the LSVC was shown to open into the LA, and an abnormal tubular structure connected to the LSVC was detected besides the LSVC–LA opening. The LSVC and IVC were connected to the LA, and the RSVC was connected to the RA. Left-sided contrast echocardiography was used in this case. The bubbles appeared in the LSVC immediately after injecting Sonovue (Bracco, Milan, Italy) into an upper extremity vein, and the bubbles appeared in the abnormal tubular structure after several cycles (Supplementary videos 3 and 4). The results suggested that the abnormal tubular structure connected to the LSVC was not the SVC and its tributary. Left-sided contrast echocardiography can help distinguish between the LSVC and CPV. The patient's anatomy was confirmed by CTA subsequently. CTA showed the left and RPVs taking a tortuous pathway into the aneurysmal LSVC, and the other lesions shown by CTA were the same as those in echocardiography.

#### 3.2.2 Cardiac TAPVC

**Figure 3:**
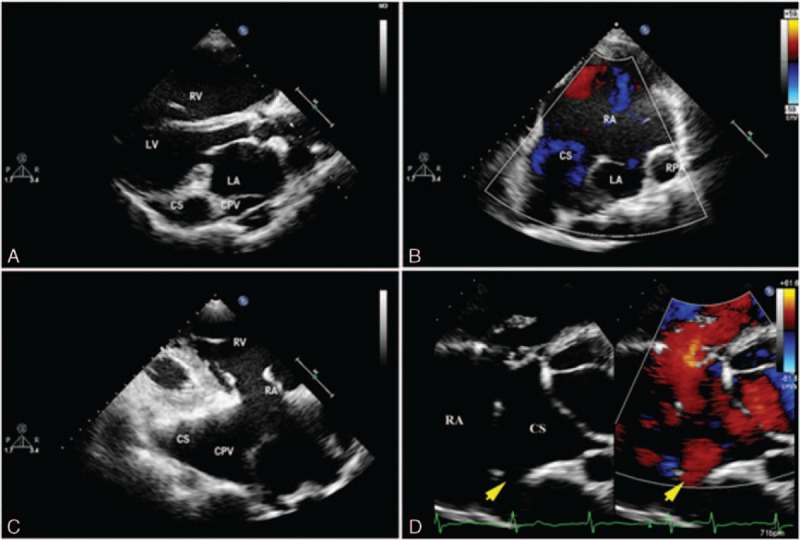
Multiple views showing the total anomalous pulmonary venous connection of a 53-year-old woman. (A) Common pulmonary vein (CPV) behind the left atrium and the enlarged coronary sinus (CS) in the left atrioventricular groove can be detected by parasternal long-axis view. (B) Anteromedial tilting bicaval view shows the CS drainage into the right atrium and atrial septal defect with right–left shunt flow. (C) CPV confluence to CS shown via the irregular right ventricular inflow tract view. (D) Flow spray to CS from CPV as shown by anteromedial tilting 4-chamber view.

In patients with the pulmonary vein connected to the CS, the dilated CS and CPV can be visualized via the parasternal view. Tracing the CS and CPV using the apical 4-chamber view and its anteromedial tilting view, the pulmonary venous confluence to the CS can be visualized. The flow into the CS can be detected by color Doppler (Fig. 3).

**Figure 4:**
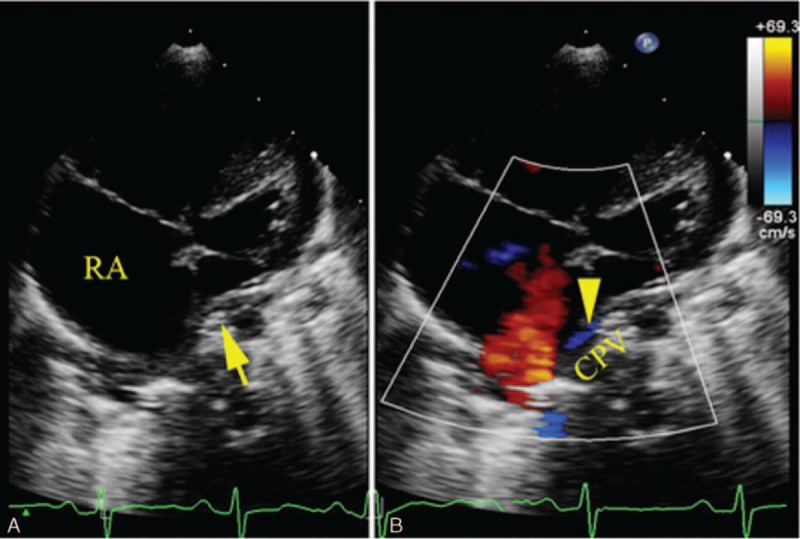
A 6-month-old boy with common pulmonary vein (CPV) draining into the right atrium (RA). (A) Apical 4-chamber view shows septum primum malposition. (B) Color Doppler shows blood flow from CPV to the RA.

In patients with direct APVs draining to the RA, it can also be visualized utilizing the apical 4-chamber view. Color Doppler can help show the flow to the RA. The characteristic changes in septum primum malposition are best shown with the apical 4-chamber view (Fig. 4).

#### 3.2.3 Infracardiac TAPVC

**Figure 5:**
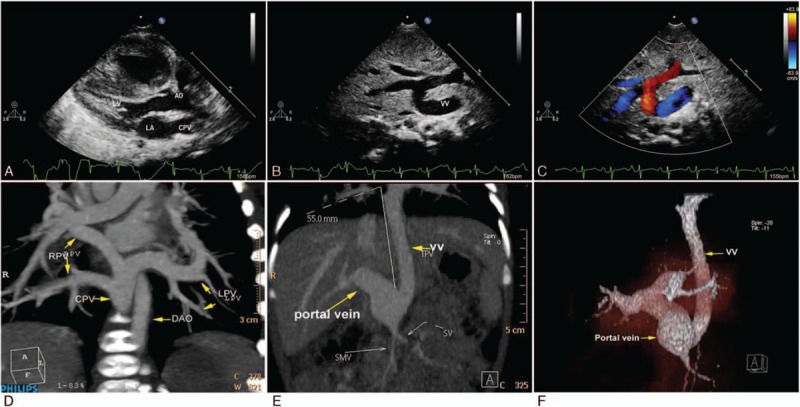
Echocardiography and computerized tomography angiography (CTA) show the infracardiac total anomalous pulmonary venous connection of a newborn baby. (A) The parasternal long-axis view shows the common pulmonary vein (CPV) in the lateral side of the left atrium. (B) The subcostal view shows the descending vertical vein (VV) draining to the portal vein. (C) Color Doppler shows the flow from VV to portal vein. (D) CTA displays the 4 pulmonary veins confluence drains into the descending VV (DVV). (E) CTA shows how the CPV traverses the diaphragm. (F) Computerized tomography volume rendering shows the DVV draining into the portal vein.

One patient with infracardiac TAPVC was identified by CTA. This patient had the CPV connected to the portal vein, and echocardiography showed that the CPV and the descending VV (DVV) drained into the portal vein (Fig. 5, Supplementary video 5). Color Doppler further showed the flow direction in the DVV to be the same as that in the descending aorta. CTA and computerized tomography volume rendering completely displayed the pathway of the APVs.

In addition, there are 10 more cases diagnosed as infracardiac TAPVC by echocardiography, but not identified by surgery or CTA. Three different drainage pathways were found including having the CPV connected to the portal vein, hepatic vein, and IVC via the descending VV. The common echocardiography features of these patients are enlargement of the right heart with a hypoplastic left heart and a dilated IVC with an accelerated flow in the IVC. Using the parasternal view, the CPV was shown in the lateral side of the LA. Next, via the subcostal view, a DVV, which traverses the diaphragm at the esophageal hiatus, was shown.

#### 3.2.4 Mixed type of TAPVC

There were 2 cases identified as the mixed type of TAPVC by surgery, but both were diagnosed as a supracardiac TAPVC by echocardiography before surgery. The pulmonary veins connected to the SVC were detected, but the other pulmonary veins connected to the RA were not detected.

### 3.3 Echocardiographic features of PAPVC

**Figure 6:**
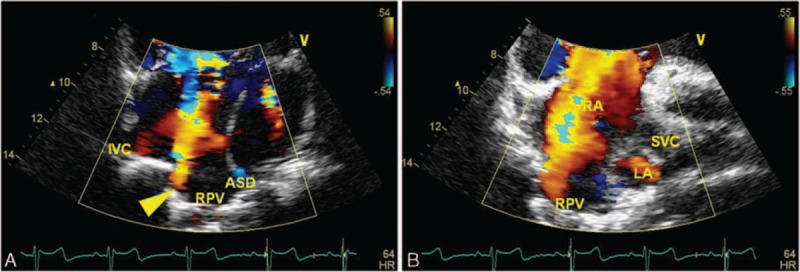
Color Doppler of the right pulmonary vein draining into the right atrium (RA) of a 13-year-old boy. (A) Apical 4-chamber view shows the right pulmonary vein (RPV) and inferior vena cava draining into the RA and the atrial septal defect located at the top of the atrial septum. (B) The apical rotation view shows the superior vena cava and RPV draining into the RA.

**Figure 7:**
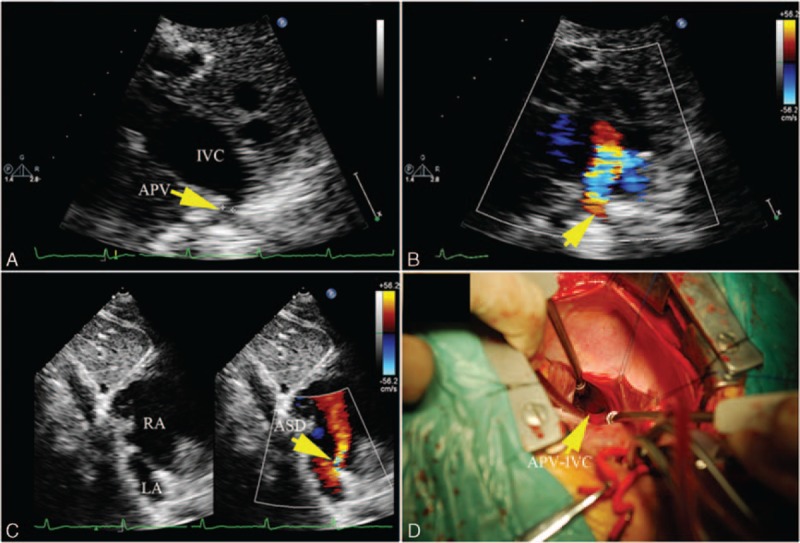
A 24-year-old girl with right pulmonary vein drainage into the inferior vena cava (IVC). (A) Subcostal view shows a tube drainage to IVC. (B) Color Doppler shows the abnormal flow spray into the IVC. (C) 2-dimensional and color Doppler showing asecundum atrial septal defect. (D) Intraoperative image shows an anomalous opening to the IVC.

A RPV anomalous connection was shown in all 33 cases of PAPVC, and most of them were associated with the SVASD (Fig. 6). Utilizing the anteromedial tilting apical 4-chamber view and the subcostal view, an abnormal flow spray into the RA could be detected in most cases. There were 2 cases where the RPV drained into the IVC below the diaphragm associated with ecundum atrial septal def. With the subcostal view, color Doppler clearly showed an abnormal flow spray into the IVC, and 2-dimensional echocardiography showed an abnormal tube connected to the IVC (Supplementary video 6). Using the apical 4-chamber view, 2 left pulmonary veins (LPVs) which connected to the LA were shown (Fig. 7). There were 2 cases where the RPV connected to the CS with echocardiography characteristics similar to the cardiac type of TAPVC drained through the CS, but flow from the LPV was detected in the LA.

### 3.4 Echocardiography features of obstruction in APV

**Figure 8:**
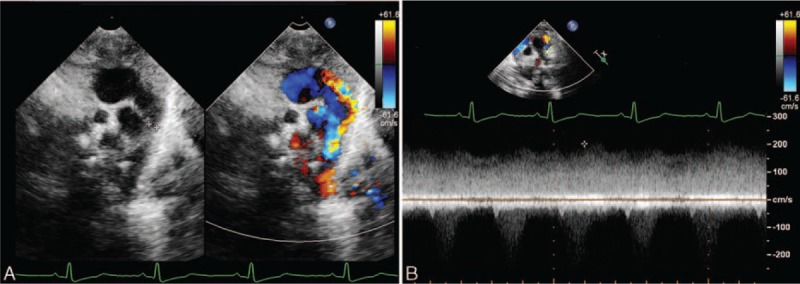
Color and pulse Doppler showing obstruction of the vertical vein in a 1-month-old girl with supracardiac total anomalous pulmonary venous connection. (A) Dual live image of 2-dimensional and flow Doppler showing turbulence flow of the obstruction. (B) Pulse Doppler shows the nonphasic flow of the stenosis.

**Figure 9:**
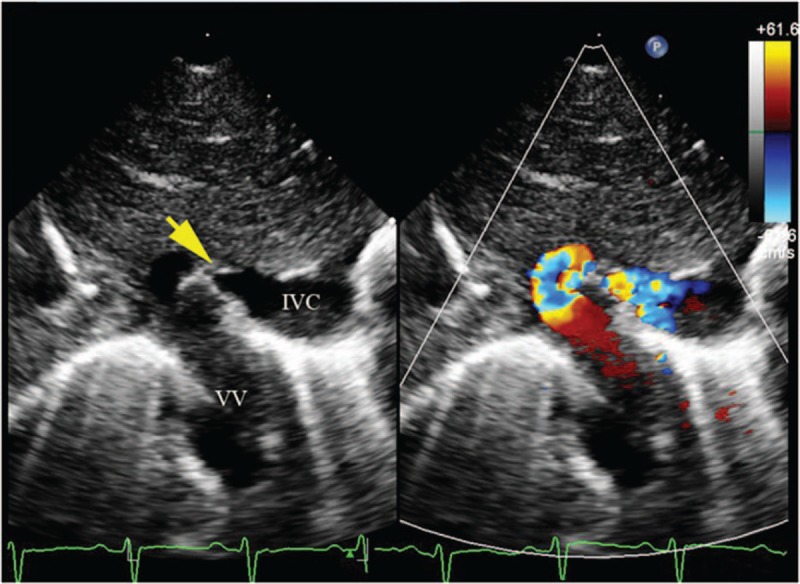
A 6-day-old baby with infracardiac anomalous pulmonary venous connection with descending vertical vein (DVV) obstruction. (A) Two-dimensional echocardiography showing the obstruction at the site of the DVV draining into the inferior vena cava, color Doppler shows the turbulence flow at this site. (B) Pulse Doppler of flow in the anomalous pulmonary vein with obstruction shown as nonphasic with high velocity.

The vessel stenosis cannot be clearly shown by 2-dimensional echocardiography. Using a combination of color flow and pulse Doppler, obstruction of the TAPVC can be observed as an accelerated flow into the stenosis with a nonphasic flow pattern (Figs. 8 and 9).

## 4 Discussion

A normal pulmonary venous system consists of 4 veins, 2 on each side, draining into the LA.^[6]^ An APVC is due to abnormalities of the development of the pulmonary veins during embryonic life. The early atresia of the CPV while pulmonary–splanchnic connections are still present results in anomalous pulmonary venous return.^[7,8]^ The APVC cases accounted for 0.7% of all the cardiac surgical series at our center for the period reviewed. The cardiac type is the most common subtype in our report, a finding that is inconsistent with findings in previous reports where the supracardiac type is more common.^[9]^ The APVs of the TAPVC more frequently drained to the LIV and the CS. The right lobe pulmonary veins more commonly drained to the RA in the PAPVCs. These findings were consistent with those of the other reports.^[1,10]^

Most of the patients with a TAPVC showed clinical symptoms within a year after birth due to the 4 pulmonary veins completely draining into the right side of the heart.^[11]^ If pulmonary vein stenosis occurs, severe dyspnea and cyanosis would appear within 1 day after birth, particularly with the infracardiac type.^[12]^ All the infracardiac cases we met by echocardiography did not undergo surgery due to severe symptoms, and 1 newborn died 6 days after birth. However, for PAPVC, most patients are asymptomatic until adulthood. In adults, PAPVC is frequently an incidental finding. Consequently, the age distribution of the 2 types was significantly different. Cases less than 1 year accounted for 75% in TAPVCs, while cases older than 10 years accounted for 47.8% of PAPVCs. One patient with cardiac TAPVC aged 53 years was the oldest in our report and also in the reported literature. The patient tolerated the symptoms because of a massive ASD. A case of a 17-year-old man diagnosed with an obstructed pulmonary vein draining to the hepatic vein was also reported.^[13]^ The presence of a large ventricular septal defect in this patient may have contributed favorably to his survival. All 82 patients were diagnosed by transthoracic echocardiography, and the sensitivity and specificity were as high as 97.5% and 99.8%, respectively, and were influenced by misdiagnosed PAPVCs. The diagnostic accuracy of classification was 93.9%, mainly due to the misdiagnosed mixed type. As a result, echocardiography showed a high accuracy in diagnosing TAPVCs and typing the supracardiac and cardiac TAPVCs, while the PAPVC and mixed TAPVC posed a challenge for echocardiography.

Key steps to enable accurate diagnosis of an APVC based on our acquired knowledge and review of the literatures.^[14–18]^ Step 1—clearly identify the existence of the APVC. APVCs should be considered when the right heart is enlarged with or without ASD, particularly in patients with an ASD. Very few cases of APVCs occur without an ASD.^[19,20]^ There was only 1 case of a PAPVC without an ASD in our study. A PAPVC may be easily overlooked in a patient with an ASD. ASD is also caused by the volume increase of the right heart. In our series, 2 cases of PAPVC were diagnosed after ASD repair surgery and a second surgery was performed to correct the APVC. In normal cases, one of the LPVs can be visualized with the parasternal long-axis view; with the apical 4-chamber view, the LPVs and the right upper lobe pulmonary vein can be shown. It is a challenge to show all 4 pulmonary veins by transthoracic echocardiography. The 2 overlooked cases of PAPVC were due to an unclear appearance of the flow from the RPV and failure to observe anomalous flow to the RA. According to our experience, a positive diagnosis can be pointed out when a PAPVC is diagnosed as ambiguous in patients with an SVASD, which is commonly associated with an upper lobe, partial anomalous pulmonary venous return.^[21,22]^ Moreover, the subcostal and suprasternal views are important to other less-common drainage pathways, including the RPV draining into the SVC, or IVC, and LPV draining into the LIV, LSVC, or CS. The 2 cases where the RPV drained into the IVC were detected over the subcostal view, a finding that may be overlooked with the apical view. We diagnosed 2 cases where the RPV connected to the CS, a finding that has been rarely reported.^[23]^

Step 2—define the drainage site and returning course. Defining the drainage site and returning course is important for surgery.^[24]^The suprasternal, subcostal, and apical 4-chamber view and its anteromedial tilting view are valuable for tracing the path of the pulmonary veins. However, the pulmonary veins can drain in numerous permutations, particularly for supracardiac TAPVCs. In this type, the connections include the LIV, which is found frequently, the SVC vein, and the AZ. An anomalous connection to the right SVC via the right VV is often associated with HS or multiple complex congenital abnormalities. An APVC to the LSVC is a rare anomaly,^[25]^ and we have reported this rare case associated with an aneurysmal LSVC. Review of the literature showed a rare case of bilateral APVC to bilateral superior caval veins.^[26]^

The cardiac type has a relatively high diagnostic accuracy, but the pulmonary veins drain separately, increasing the difficulty in making an accurate diagnosis. The case inaccurately diagnosed by echocardiography, in which the LPV was misdiagnosed as a VV, belongs to this type and will be discussed.

The infracardiac type diagnosis should be doubted if the common features of a TAPVC appear but the supracardiac and cardiac type have been excluded. A descending VV, dilated hepatic vein, and IVC visualized over the subcostal view suggest the infracardiac type. The mixed type has the lowest diagnostic accuracy in our report and in previous reports.^[4,27]^

**Figure 10:**
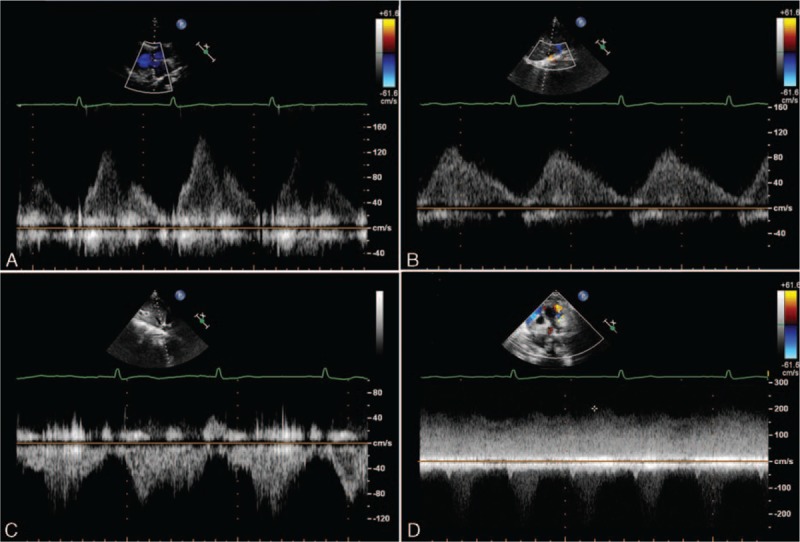
Pulse Doppler of different patterns in pulmonary veins, anomalous pulmonary veins (APVs), obstructed APVs, and systemic veins. (A) Pulse Doppler of phasic flow in the left pulmonary vein, varying with the cardiac cycle. (B) Pulse Doppler of phasic flow in the APV, varying with the cardiac cycle. (C) Pulse Doppler of nonphasic flow in the inferior vena cava with low velocity. (D) Pulse Doppler of APV with an obstruction, nonphasic flow with high velocity.

Step 3—examine for the existence of obstruction in the pathway. Pulse Doppler showed different patterns in APVs, obstructed APVs, and systemic veins. Flow in the pulmonary veins without obstruction was phasic, varying with the cardiac cycle, which is different from obstructed veins and systemic veins. In those with venous obstruction, the flow was nonphasic, varying only with respiration. In systemic veins, the flow was nonphasic and with low velocity (Fig. 10).

Whether an obstruction exists in the pulmonary vein is critical for the timing of surgery, particularly for the infracardiac type of TAPVC, which is most likely to merge obstruction.^[28]^ The obstruction may occur at any point along the anomalous path, including the esophageal hiatus, the portal venous system, the ductus venous, or at the level of the hepatic sinusoids.^[8]^ A previous study has also reported that about 50% of the supracardiac connections were obstructed, and the most common sites of obstruction were in the left VV at the level of the left pulmonary artery and in the right VV at its insertion into the SVC.^[29]^ Three cases with obstruction in the supracardiac TAPVC were identified. Two obstructions were in the left VV, and 1 obstruction was at the insertion to the RSVC. The turbulent flow in the pathway as shown by color Doppler and the nonphasic flow as shown by Pulse Doppler in the narrow site are the key points of diagnosing an obstruction.

**Figure 11:**
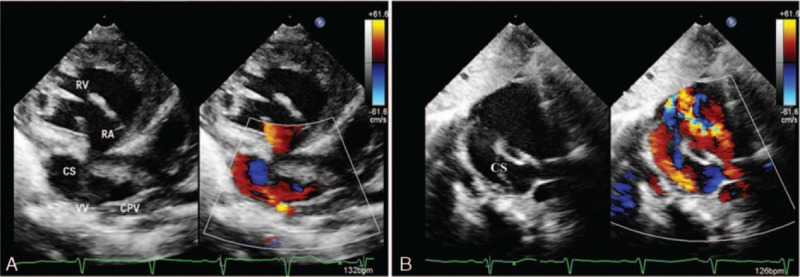
Echocardiography misdiagnosed the drainage pathway of a 3-month-old boy. (A) The irregular apical 4-chamber view shows the pathway of common pulmonary vein–vertical vein (VV)–coronary sinus (CS), the VV was identified as a narrowed left pulmonary vein by surgery. (B) The subcostal view showed a flow to CS.

The drainage pathway of 1 case with cardiac TAPVC was misdiagnosed. The case was diagnosed as TAPVC in which CPV was connected to the CS via an obstructive VV by echocardiography (Fig. 11, Supplementary video 7). The VV was identified as the narrowed LPV in surgery, and the left and RPVs drained into the CS separately. Reviewing the ultrasound images, the RPV draining into the CS was not observed because of the short path and a single anomalous flow detected in the CS. This case suggests that multiple variation of APVs increases the difficulty in ultrasound diagnosis.

Five cases without PAPVCs identified by surgery or CTA were misdiagnosed by echocardiography, 4 cases of which were associated with an SVASD. Reviewing the images of these cases revealed 2 cases in which RPVs cannot be detected clearly. The other 2 cases had defects on the roof of atrial septal and close to the opening of RPV. Color Doppler showed the flow from RPV spray into the RA through the ASD, resulting in the misdiagnosis of PAPVC. Surgery identified that the RPV opening was located on the LA beside the atrial septum.

**Figure 12:**
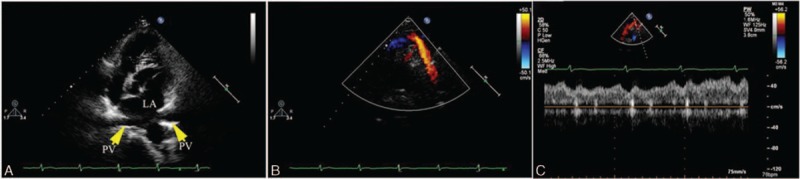
A case misdiagnosed as supracardiac partial anomalous pulmonary venous connection. (A) Two openings of the pulmonary vein into the left atrium can be detected clearly by apical view. (B) An upward venous drainage to left innominate vein as shown via suprasternal view, which was identified as anomalous azygos vein by computerized tomography angiography. (C) Pulse Doppler of this anomalous vein showing low velocity and nonphasic flow.

The other case was first diagnosed as a single pulmonary vein draining to the LIV via the VV (Fig. 12). However, CTA showed this case to be rare AZ drainage to the LIV. Reviewing the echocardiography images, detection of upward venous drainage to the LIV and the lack of showing all 4 openings in the atrium compelled us to doubt a PAPVC of the supracardiac type (Supplementary video 8). However, this patient had a normal-sized chamber, and an ASD or PFO was not detected, findings that are inconsistent with the common features of APVC. Meanwhile, the pulse Doppler of this anomalous vein performs as low velocity and nonphasic flow did not consist with the characteristic of APV. This case suggests that venous drainage to the LIV may be its anomalous tributary.

Although CTA and multidetector computed tomography have significant advantages in accurately evaluating the course and the number of anomalous veins, ultrasound diagnosis is the first-line choice for diagnosis of congenital heart disease because it is noninvasive, inexpensive, and repeatable.^[30,31]^ Technological advances in echocardiography equipment have made identification and classification of APVC more accurate by following certain critical steps. Echocardiography can provide a definite pathway of pulmonary veins in typical APVC types but markedly limited information in the atypical type and the mixed type. Further examination should be carried out to gain more information.

There are a few limitations related to this study. First, not all the 84 APVCs underwent surgical correction, 2 cases of which were confirmed by CTA. Although angiography is the gold standard for evaluation, it carries certain inherent risks, especially in small and sick infants. Literatures have reported that CTA allows detailed and comprehensive evaluation of the APVC. Second, few ultrasound techniques were used for evaluation of APVC, including 2-dimensional echocardiography and Doppler echocardiography. Future advances may focus on other techniques such as transesophageal echocardiography to improve diagnostic accuracy.

## Acknowledgments

The authors thank John Lof for his assistance in preparing this manuscript.

## Supplementary Material

Supplemental Digital Content

## Supplementary Material

Supplemental Digital Content

## Supplementary Material

Supplemental Digital Content

## Supplementary Material

Supplemental Digital Content

## Supplementary Material

Supplemental Digital Content

## Supplementary Material

Supplemental Digital Content

## Supplementary Material

Supplemental Digital Content

## Supplementary Material

Supplemental Digital Content
